# A novel inducible prophage from the mycosphere inhabitant *Paraburkholderia terrae* BS437

**DOI:** 10.1038/s41598-017-09317-8

**Published:** 2017-08-22

**Authors:** Akbar Adjie Pratama, Jan Dirk van Elsas

**Affiliations:** Department of Microbial Ecology, Microbial Ecology - Groningen Institute for Evolutionary Life Sciences, University of Groningen, Nijenborgh 7, Groningen, 9747 AG The Netherlands

## Abstract

Bacteriophages constitute key gene transfer agents in many bacteria. Specifically, they may confer gene mobility to *Paraburkholderia* spp. that dwells in soil and the mycosphere. In this study, we first screened mycosphere and bulk soils for phages able to produce plaques, however found these to be below detection. Then, prophage identification methods were applied to the genome sequences of the mycosphere-derived *Paraburkholderia terrae* strains BS001, BS007, BS110 and BS437, next to *P*. *phytofirmans* strains BS455, BIFAS53, J1U5 and PsJN. These analyses revealed all bacterial genomes to contain considerable amounts [up to 13.3%] of prophage-like sequences. One sequence predicted to encode a complete phage was found in the genome of *P*. *terrae* BS437. Using the inducing agent mitomycin C, we produced high-titered phage suspensions. These indeed encompassed the progeny of the identified prophage (denoted ɸ437), as evidenced using phage major capsid gene molecular detection. We obtained the full sequence of phage ɸ437, which, remarkably, had undergone a reshuffling of two large gene blocks. One predicted moron gene was found, and it is currently analyzed to understand the extent of its ecological significance for the host.

## Introduction

Viruses that infect bacteria - bacteriophages (phages) - play significant roles in the evolution of bacteria, at both the individual and community levels^1^. As agents of horizontal gene transfer (HGT), phages can enhance the fitness of their host cells in the form of lysogenic conversion and/moron genes, for instance by providing so-called auxiliary metabolic genes (AMGs)^2^ as well as virulence or pathogenicity traits^3^. Moreover, phages function in the biological ‘warfare’ among neighboring bacterial cells and can modulate the formation of bacterial biofilms at the population level^4^.

Prophages - temperate phages that occur in an integrated form in the bacterial genome - are often present in considerable amounts in bacterial genomes. For example, a recent study^5^ of 69 *Escherichia* and *Salmonella* genomes revealed prophages to occupy up to 13.5% of the genome of *Escherichia coli* O157:H7 [strain EC4115] and up to 4.9% of that of *Salmonella* Newport strain SL254. Such prophages, when intact, may be induced from the host genome, yielding phage progeny in lysates. This may occur as a response to stress, for instance resulting from exposure to UV^6, 7^, hydrogen peroxide^8^ or mitomycin C (MMC)^9^. Moreover, prophages can be ‘spontaneously’ induced, which implies that cues of unknown nature may have been at the basis of induction^10^. However, many potential prophages are, to different extents, defective or ‘cryptic’, as they have been subjected to genetic erosion (degradation and deletion) processes^11–13^. Such defective prophages may endow their hosts with gene repertoires that allow survival in harsh environments^14^.

The extant abundance of phages, as compared to their bacterial hosts, is often astounding^12, 15^. However, we have so far only just scratched the ‘tip of the phage iceberg’. Moreover, whereas most studies on phages have been made in aquatic ecosystems^2, 16–18^, those in soil have been lower in number or have only just emerged^19, 20^.

Members of the genus *Burkholderia* exhibit a tremendous phenotypic diversity and they inhabit diverse ecological settings^21^, ranging from soil^22, 23^ to plants and humans^24^. A recent study divides *Burkholderia* into two clades, in which clade I contain all pathogenic *Burkholderia* species and clade II mainly so-called “environmental” bacteria. Clade II was renamed *Paraburkholderia* ^21, 25^. This genus encompasses members with the largest genomes among all known bacteria. Such genomes may have resulted from frequent HGT events and potential selection^26^. Zhang *et al*.^27^ recently provided arguments for the tenet that the mycosphere, in the light of the bacterium-‘feeding’ fungus and the multitude of active bacteria occurring there, constitutes a true arena that fosters HGT. Hence, there is great interest in digging deeper into the genetic legacies of such events in mycosphere dwellers. Nazir *et al*.^28^ described a suite of truly fungal-interactive *Paraburkholderia* strains, including *P*. *terrae* strains BS001, BS007, BS110 and BS437, and *P*. *phytofirmans* BS455. Analysis of the 11.5 Mb large genome of the then selected *P*. *terrae* BS001 - in comparison with other similar genomes - revealed 96% of it to belong to the non-core [variable] part^26^. Some evidence was presented for the presence of phage-typical integrases, along with other phage-related genes, raising the question whether phages could facilitate HGT in this organism.

In this study, we hypothesized that prophage sequences present in some of the aforementioned fungal-interactive *Paraburkholderia* strains can give rise to phage populations that foster adaptive processes in *Paraburkholderia* in the mycosphere. We thus first screened the mycosphere (and corresponding bulk soil) for free phages and then – in a search for prophages – examined the genomes of mycosphere-derived *Paraburkholderia* strains. Indeed, evidence was found for the presence of putative prophage and phage-like elements in several genomes. We then focused on a predicted full-phage sequence found in *P*. *terrae* strain BS437, the data of which are presented here. To the best of our knowledge, this is the first study that isolates induced prophage from *Paraburkholderia* isolated from the mycosphere.

## Results

### Screening of mycosphere and bulk soil samples for free *Paraburkholderia* phages

Given the fact that previous studies^28, 29^ revealed a prevalence of *Paraburkholderia* types (in particular *P*. *terrae*) in the mycospheres of different soil fungi, we first screened two freshly-sampled mycospheres (*Scleroderma citrinum* and *Galerina* spp.) for the presence of phage particles able to produce plaques on selected strains of *Paraburkholderia* spp. including *P*. *terrae*, *P*. *phytofirmans*, *P*. *caribensis*, *P*. *hospita* and *P*. *terricola* (for details of the strains, see Table S1). Both direct extracts and fivefold phage-enriched ones (See Materials and Methods) were tested. This first attempt to detect phages that, in a lytic or temperate manner, productively interact with any of the selected *Paraburkholderia* species was done using the classical double-agar-layer [DAL] method^30^ and spot tests. Unfortunately, neither the crude phage preparations from the mycosphere as well as bulk soil samples nor the phage enrichments showed any single plaques or lysis zones across all assays that were performed. This indicated an insufficiently low titer of virions in the extracts that were able to produce detectable clear or turbid plaques on the lawns of indicator bacteria used (Table S1).

### Analysis of putative prophage regions across *Paraburkholderia* genomes

In the light of the presumed low prevalence of free phage particles in the mycosphere as well as bulk soil samples, we then examined the putative presence of integrated phage. For that, we analyzed the genomes of the mycosphere-derived *P*. *terrae* strains BS001, BS007, BS110 and BS437, as well as of *P*. *phytofirmans* strains BS455, BIFAS53, J1U5 and PsJN, for the presence of putative prophage-like (PP) elements (Table S2). For this, we used the phage identification programs PHAST^31^, Prophinder/ACLAME^32^ and PhiSpy^33^. By applying the criteria (see Material and Methods), we identified a total of 209 PP regions across the eight *Paraburkholderia* genomes. Following curation, 127 of the regions remained for further analyses (Tables 1 and S2). Most of these predicted prophage regions (Table S2) were interpreted as putative legacies of previous phage insertions, as they appeared to have lost essential phage core genes^13^.

**Table 1:** Genome ɸ437 assignment.

ORF	−/+	start	stop	aa	RAST annotation function	PSI-BLASTP best hit (gene)[Taxa]	Cov. (%)	E-value	Id. (%)	Acc. Blast hit
1	**−**	1372	779	198	Hypothetical protein	Hypothetical protein AcaML1_0023 [*Acidithiobacillus* phage AcaML1]	38	0,00005	33	AFU62868
2	**−**	1553	1410	48	Hypothetical protein	Phage protein gp26 [*Burkholderia* phage BcepB1A]	59	4.4	32	YP_024873
3	**−**	2233	1550	228	Hypothetical protein	Minor tail protein [*Rhodobacter* phage RcRhea]	94	1E-22	33	YP_009213512
4	**−**	2505	2233	91	Hypothetical protein	Hypothetical protein TAEYOUNG_67 [*Arthrobacter* phage TaeYoung]	72	1.4	29	ALY10524
5	**−**	2874	2749	42	Hypothetical protein	Virion encapsidated RNAP [*Erwinia* phage vB_EamP-S6]	78	0.77	41	YP_007005815
6	+	3126	3335	70	Hypothetical protein	Endolysin [*Erwinia* phage vB_EamM-Y2]	73	7.0	25	YP_007004738
7	+	3658	3909	84	Hypothetical protein	Hypothetical protein FV3_00119 [*Escherichia* phage FV3]	51	0.84	37	YP_007006290.
8	+	3899	4318	140	Hypothetical protein	Plasmid stability protein [*Synechococcus* phage S-SSM5]	23	4.5	47	YP_004324760
9	**−**	4991	4383	203	Hypothetical protein	Hypothetical protein SEA_VINCENZO_40 [*Mycobacterium* phage Vincenzo]	22	1.5	31	YP_009210896
10	**−**	5242	4988	85	Phage protein	Hypothetical protein Bcep22_gp19 [*Burkholderia* virus Bcep22]	89	4E-21	49	NP_944247
11	**−**	5592	5239	118	Hypothetical protein	Hypothetical protein BcepF1.080 [*Burkholderia* virus BcepF1]	36	1.8	35	YP_001039764
12	**−**	5855	5592	88	Phage protein	Phage protein gp3 [*Burkholderia* phage Bcep176]	100	3E-22	45	YP_355338
13	**−**	6325	5852	158	Hypothetical protein	Phage conserved protein gp66 [*Burkholderia* virus phi1026b]	81	1E-38	56	NP_945097
14	**−**	6639	6322	106	Hypothetical protein	Hypothetical protein DM_180 [*Erwinia* phage vB_EamM_Deimos-Minion]	45	0.42	35	ANH52278
15	**−**	6993	6658	112	Phage protein	Hypothetical protein Ac42p014 [*Acinetobacter* phage Ac42]	100	3E-20	45	YP_004009376
16	**−**	7462	6977	162	Hypothetical protein	Phage protein gp74 [*Burkholderia* virus phi1026b]	86	7E-52	58	NP_945105
17	**−**	7904	7542	121	Phage protein	Unnamed protein product [*Pseudomonas* phage phi297]	86	2E-29	47	YP_005098034
18	**−**	8551	7901	217	Hypothetical protein	Hypothetical protein DIBBI_075 [*Xanthomonas* phage vB_XveM_DIBBI]	17	0.55	34	YP_006383682
19	**−**	9150	8548	201	Phage Holliday junction resolvase	Putative endodeoxyribonuclease RusA [*Burkholderia* phage Bups phi1]	73	2E-45	54	ABY40522
20	**−**	10331	9147	395	Replication protein O	DNA replication protein [*Salmonella* phage vB_SemP_Emek]	41	3E-14	31	YP_006560599
21	**−**	10586	10332	85	Hypothetical protein	DNA-binding protein [*Caulobacter* phage Sansa]	50	0.079	38	AKU43488
22	**−**	10894	10583	104	Hypothetical protein	Hypothetical protein QHH_02 [*Halomonas* phage QHHSV-1]	33	0.027	47	APC45914
23	**−**	11123	10911	71	Hypothetical protein	Tail component protein gp17 [*Burkholderia* phage KS9]	88	0.009	35	YP_003090193
24	**−**	11450	11259	64	Hypothetical protein	Putative HNH endonuclease [*Brucella* phage 02_19]	28	3.1	56	AKO58996
25	**−**	11847	11452	132	Hypothetical protein	Transcriptional regulator [*Staphylococcus* phage IME-SA4]	22	0.24	47	YP_009219655
26	**−**	12239	11952	96	Hypothetical protein	Hypothetical protein [*Moraxella* phage Mcat7]	74	0,00000005	32	AKI27330
27	+	12457	13317	287	Phage repressor	Phage CI repressor [*Bacteriophage* APSE-2]	48	6E-28	45	YP_002308514
28	+	13702	13857	52	Hypothetical protein	Putative tape measure protein [*Gordonia* phage GMA3]	45	0.45	48	YP_009188584
29	+	13857	14108	84	Hypothetical protein	Hypothetical protein BPS10C_040 [*Bacillus* phage BPS10C]	22	4.6	37	YP_009002926
30	+	14105	14254	50	Hypothetical protein	Phage protein gp41 [*Burkholderia* phage Bcep176]	100	0,0002	42	YP_355376
31	+	14254	14379	42	Hypothetical protein	Tail fibers protein [*Escherichia* phage 64795_ec1]	43	0.31	61	YP_009291518
32	+	14582	14914	111	Phage protein	Hypothetical protein BcepIL02_gp10 [*Burkholderia* virus Bcepil02]	90	9E-14	38	YP_002922682.
33	+	14961	15794	278	Hypothetical protein	Hypothetical protein BcepIL02_gp11 [*Burkholderia* virus Bcepil02]	96	6E-50	36	YP_002922683
34	+	15870	16580	237	Phage-related protein	Hypothetical protein F116p07 [*Pseudomonas* phage F116]	99	5E-47	45	YP_164271
35	+	16577	16912	112	Hypothetical protein	Hypothetical protein DC1_00025 [*Burkholderia* virus DC1]	74	0.001	30	YP_006589955
36	+	16991	17842	284	Hypothetical protein	Hypothetical protein BcepF1.035 [*Burkholderia* virus BcepF1]	21	0.89	40	YP_001039719
37	+	17873	18127	85	Hypothetical protein	Hypothetical protein [*Enterobacteria* phage P2-EC31]	89	0,0001	32	CAJ43161
38	+	18129	18605	159	Hypothetical protein	Phage protein gp42 [*Burkholderia* virus phi1026b]	29	2.1	30	NP_945073
39	**−**	18802	18608	65	Hypothetical protein	Endolysin [*Arthrobacter* phage Gordon]	46	8.2	37	ALY08979
40	**−**	19078	18821	86	Hypothetical protein	hypothetical protein PBI_ZAPNER_53 [*Mycobacterium* phage Zapner]	31	0.97	41	AHZ95507
41	**−**	19393	19205	63	Hypothetical protein	Hypothetical protein SPN3US_0221 [*Salmonella* phage SPN3US]	38	1.2	48	YP_009153515
42	+	19465	19938	158	Hypothetical protein	**−**	**−**	**−**	**−**	**−**
43	+	19976	20923	316	Bacteriophage protein gp37	Hypothetical protein gp38 [*Burkholderia* virus phi1026b]	100	4E-137	62	NP_945069
44	**−**	21318	20968	117	Hypothetical protein	Hypothetical protein [EBPR siphovirus 1]	55	0.11	30	AEI71224
45	+	21353	21742	130	Hypothetical protein	Hypothetical protein Bcep22_gp48 [*Burkholderia* virus Bcep22]	81	6E-29	52	NP_944277
46	**−**	21956	21696	87	Hypothetical protein	Unnamed protein product [*Bacillus* phage SPP1]	30	9.2	35	NP_690702
47	+	22248	23240	331	phage integrase family protein	Integrase [*Pseudomonas* phage D3]	40	2E-12	33	NP_061531
48	**−**	23786	23520	89	Hypothetical protein	Major capsid protein [uncultured *Myoviridae*]	60	0.76	32	ACT78915
49	**−**	24491	23805	229	protein of unknown function DUF159	Hypothetical protein gp28 [*Burkholderia* phage KS9]	90	8E-57	45	YP_003090205
50	**−**	25032	24541	164	Hypothetical protein	Hypothetical protein PBI_JAY2JAY_59 [*Streptomyces* phage Jay2Jay]	29	0.45	33	YP_009225784
51	**−**	25607	25047	187	Hypothetical protein	Baseplate hub subunit and tail lysozyme protein [*Escherichia* phage Lw1]	31	0.29	26	YP_008060715
52	**−**	26014	25625	130	Hypothetical protein	Hypothetical protein fHeYen901_253 [Yersinia phage fHe-Yen9-01]	30	0.21	41	ARB06026
53	+	26247	26951	235	RecA/RadA recombinase	Baseplate wedge subunit [*Synechococcus* phage S-RSM4]	24	5.0	31	YP_003097386
54	+	26999	27214	72	Hypothetical protein	Hinge connector of long tail fiber proximal connector [*Citrobacter* phage Merlin]	85	3.4	28	YP_009203991
55	+	27186	27833	216	LigD, ATP-dependent DNA ligase	ATP-dependent DNA ligase [*Bacillus* phage phi3T]	93	2E-20	30	APD21266
56	**−**	28268	27888	127	Tail fiber assembly protein	Hypothetical protein [*Salmonella* phage IME207]	75	2E-21	45	YP_009322735
57	**−**	28702	28268	145	Hypothetical protein	Tail protein [*Bacillus* phage BigBertha]	49	3.2	32	YP_008771129
58	**−**	29029	28745	95	Hypothetical protein	TreK [*Staphylococcus* phage phiIPLA-C1C]	32	4.5	26	YP_009214605
59	**−**	29469	29026	148	Hypothetical protein	HNH nuclease [*Bacillus* phage AR9]	95	2E-27	37	YP_009282937
60	**−**	29973	29629	115	Hypothetical protein	Hypothetical protein RcapMu34 [*Rhodobacter* phage RcapMu]	85	7E-30	57	YP_004934677
61	**−**	30422	29970	151	Chain A, D20c mutant of T4 lysozyme	Phage putative lysozyme [*Idiomarinaceae* phage Phi1M2-2]	94	2E-25	40	YP_009104271
62	**−**	30672	30424	83	Hypothetical protein	Minor tail protein Z [*Enterobacteria* phage mEp237]	31	0.79	42	YP_009224009
63	**−**	31444	30974	157	Hypothetical protein	Arc domain-containing protein [*Pseudomonas* phage PaBG]	30	2E-10	54	YP_008433620
64	+	31579	31758	60	Hypothetical protein	Putative Arc protein [*Pseudomonas* phage SM1]	79	5E-10	55	ALT58107
65	+	31813	32682	290	Phage antirepressor protein	Putative antirepressor protein Ant [*Edwardsiella* phage GF-2]	71	1E-39	39	YP_009126626
66	+	32682	33413	244	Phage DNA binding protein Roi	Putative DNA binding protein Roi [*Pseudomonas* phage PAN70]	35	8E-37	71	AIX12494
67	+	33589	34266	226	Hypothetical protein	Hypothetical protein CL2_12 [*Lactobacillus* phage CL2]	9	6.7	57	YP_009201807
68	**−**	35093	34269	275	Conserved domain protein	Glycosyl transferase [*Synechococcus* phage S-CRM01]	16	0.16	40	YP_004508523
69	**−**	35344	35156	63	Hypothetical protein	Hypothetical protein Syn7803US105_79 [*Synechococcus* phage ACG-2014g]	43	1.3	44	YP_009133639.
70	**−**	36414	35356	353	Prophage long tail fiber protein	Putative tail protein [*Burkholderia* phage Bups phi1]	76	8E-60	48	ABY40547
71	**−**	37019	36423	199	Prophage tail protein	Tail protein [*Shigella* phage SfIV]	97	1E-25	35	YP_008766883
72	**−**	38189	37026	388	Phage FluMu protein gp47	Baseplate protein [*Shigella* phage SfIV]	89	7E-34	32	YP_009147467
73	**−**	38637	38191	149	Bacteriophage protein GP46	Putative tail protein [*Salmonella* phage ST64B]	80	2e-21	42	NP_700393
74	**−**	39159	38641	173	Prophage baseplate assembly protein V	Putative base plate assembly protein [*Salmonella* phage ST64B]	87	9E-36	41	NP_700392
75	**−**	40355	39204	384	Prophage tail protein	Putative tail protein [*Escherichia* virus Mu]	89	2E-30	27	NP_050648
76	**−**	41917	40355	521	Phage tail length tape-measure protein	Phage protein gp14 T [*Burkholderia* phage BcepB1A]	28	0,000003	28	YP_291174
77	**−**	43357	41933	475	Phage tail/DNA circulation protein	Tail/DNA circulation protein [*Shigella* phage SfIV]	92	2E-36	28	YP_008766878
78	**−**	44081	43521	187	Putative phage protein	Hypothetical protein AcaML1_0057 [*Acidithiobacillus* phage AcaML1]	34	0.34	30	AFU62902
79	**−**	44459	44085	125	Phage tail tube protein	Tail tube protein [*Salmonella* phage ST64B]	91	0,00003	24	NP_700387
80	**−**	46013	44523	497	Bacteriophage tail sheath protein	Tail sheath protein [*Enterobacteria* phage SfI]	100	3E-131	42	YP_009147459
81	**−**	46198	46010	63	Mu-like prophage FluMu protein GP38	Hypothetical protein [*Escherichia* phage D108]	85	0,000007	43	YP_003335786
82	**−**	46808	46209	200	Hypothetical protein	Terminase [*Mycobacterium* phage DarthPhader]	30	7.4	28	AOZ61253
83	**−**	47142	46801	114	Putative phage protein	hypothetical protein AcaML1_0040 [*Acidithiobacillus* phage AcaML1]	84	5e-06	33	AFU62885
84	**−**	48206	47142	355	Phage-related functions and prophages	Major capsid [*Aurantimonas* phage AmM-1]	99	4E-65	38	YP_009146944
85	**−**	49229	48300	310	Hypothetical protein	Head decoration protein D [*Aurantimonas* phage AmM-1]	16	0,00004	44	YP_009146943
86	**−**	49876	49259	206	Putative phage protein	Internal virion protein D [*Pseudomonas* phage phiPsa17]	52	2.6	30	AKG94384
87	**−**	50781	49903	293	Head-tail preconnector protein GP5	Prohead protease; 36 K type signal peptide peptidase SppA [*Achromobacter* phage phiAxp-2]	76	2E-53	43	YP_009226433
88	**−**	52466	50778	563	Phage portal protein	Portal protein [*Xylella* phage Sano]	90	5E-86	35	AHB12085
89	**−**	52711	52466	82	Hypothetical protein	Phi92_gp071 [*Enterobacteria* phage phi92]	66	2.1	24	YP_009012402
90	**−**	53969	52719	417	Phage terminase, large subunit	Packaging terminase large subunit gpA [*Acidithiobacillus* phage AcaML1]	81	9E-79	44	AFU62879

Across the *P*. *terrae* strains, *P*. *terrae* BS007 had the largest (11.8%), and *P*. *terrae* BS001 the lowest (8.17%) total amount of PP region. *P*. *terrae* strain BS007 also harbored the largest PP (encoded ɸ007-5), of about 205.2 Kb. For the *P*. *phytofirmans* strains examined, *P*. *phytofirmans* J1U5 had the largest (13.4%) and *P*. *phytofirmans* BIFAS53 the lowest (2.7%) total amount of PP region. *P*. *phytofirmans* strain J1U5 harbored the highest PP number, i.e. 27. In contrast, *P*. *phytofirmans* PsJN only carried two identifiable PP regions, i.e. (encoded by us) ɸPsJN-2 (63.1 Kb) and ɸPsJN-3 (15.7 Kb). *P*. *phytofirmans* strain BIFAS53 contained the smallest identifiable PP (ɸBIFAS53-4), of about 10.5 Kb (Table S2).

For the next phase of this study, (1) only complete phage regions that could be predicted to form phage progeny, and (2) were consistently detected by all three programs, were further analyzed. It should be noted here that both PHAST and PhiSpy indicated the presence of one complete prophage in each of *P*. *phytofirmans* BS455 and PsJN. These regions however were excluded, as we placed a focus on the fungal-interactive *Paraburkholderia terrae*. Very convincingly, all three programs indicated that one full PP region was present in *P*. *terrae* BS437, with size of about 43.6 Kb (positions 6888478 to 6932098); this prophage, tentatively denoted as ɸ437, thus formed the focus of the next parts of this study.

### Bacteriophage induction in *P*. *terrae* BS437

**Figure 1:**
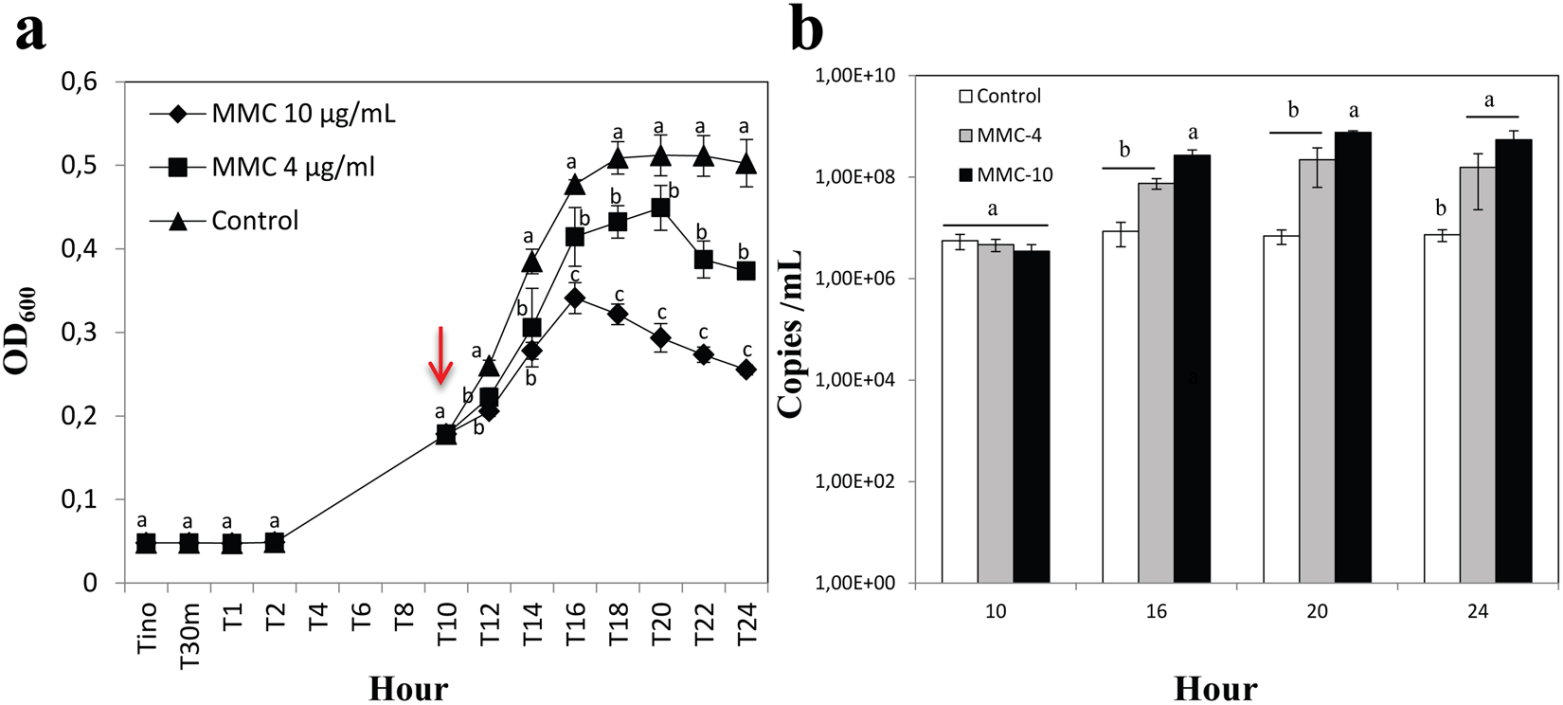
**(a)** Prophage induction and **(b)** quantitative PCR of the progeny. The MMC was added with a different concentration, MMC-4 (4 µg/mL) (■), MMC-10 (10 µg/mL) (♦) and without MMC/control (▲) to exponential-growing cell (10 hour; indicated with red arrow) of incubation in LB medium at 28 °C. Sample from 10 hour, 16 hour, 20 hour and 24 hour were used for quantitative PCR, error bars indicated s.d. values (*n* = 3). Significant of the treatments are marked with letter (**a**,**b**) for *P* < 0.05.

Given the finding of the ɸ437 encoding sequence in the *P*. *terrae* BS437 genome, cultures of this organism were screened for the presence of virions, using induction with different levels of MMC, in comparison to a control (to address spontaneous release; Fig. 1). We took a significant decrease of the OD_600_ in the BS437 cultures, following addition of MMC, as an indication that prophage had been induced to excise from the host genome, resulting in production of enhanced levels of phage progeny. Indeed, MMC had a population-reducing effect, as measured by the OD_600_ of the cultures, with higher levels of MMC resulting in stronger decreases of the OD_600_. Specifically, mid-log-phase cultures - upon treatment with 10 μg/mL MMC - showed significant decreases (ANOVA *n* = 3, *P* < 0.05) of the OD_600_ as compared to the untreated control up to 14 h. In the control, at 10 h, exponential growth was found, with the stationary phase at 18 h being followed by a slow decrease of optical density at 24 h (Fig. 1a).

**Figure 2:**
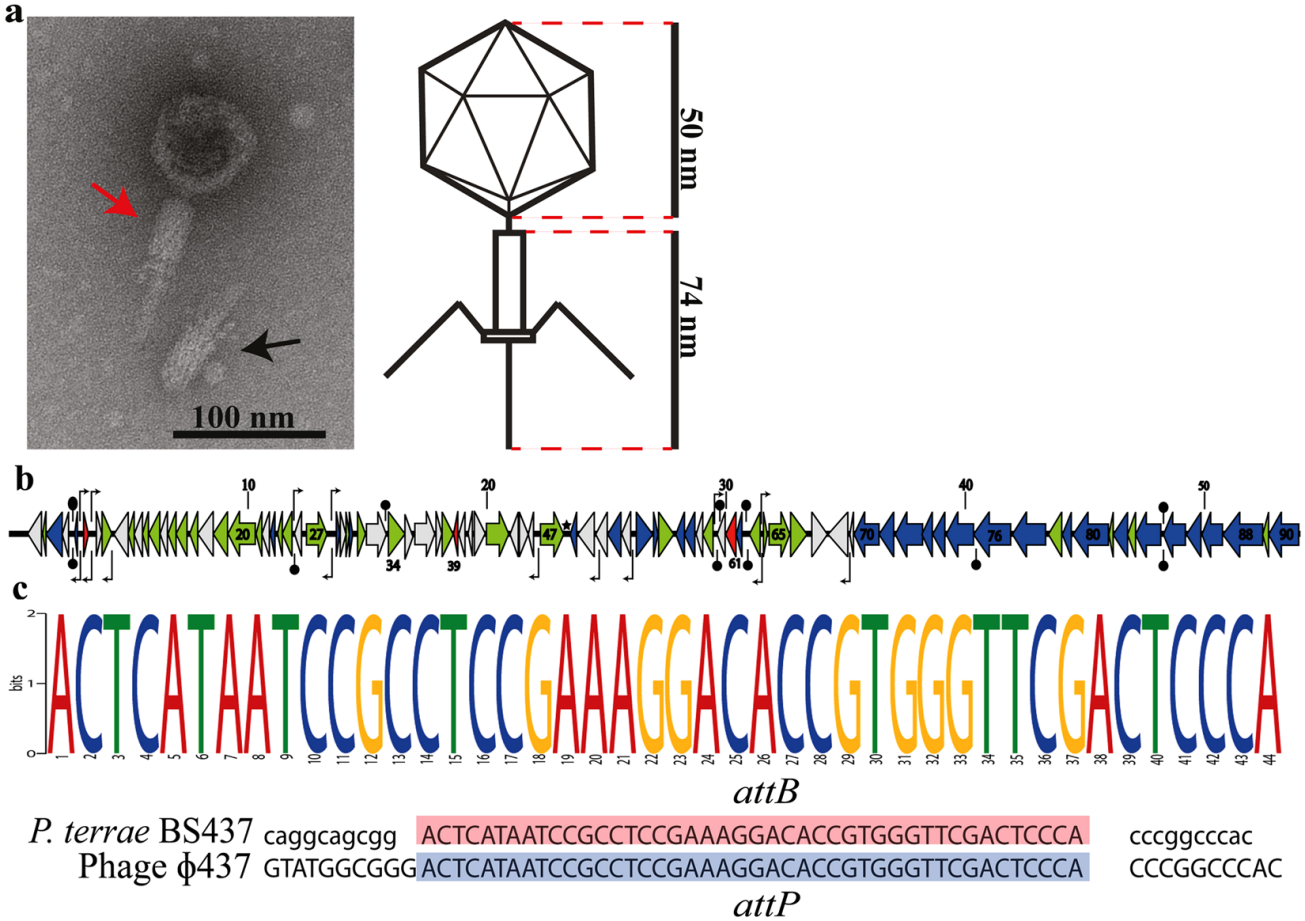
**(a)** The TEM image and approximate induced prophage measurement. Crude induced lysate was filtered with 0.22-µm-pore-size filter and centrifuged to pellet the cell derbies, then store in −20 °C for one night prior imaging. Image shows a typical *Myoviridae* family, the image also shows induced ɸ437 (red arrow) and ɸ437 that has lost its capsid structure (black arrow). The bar represents 100 nm. **(b)** genome sequence of ɸ437. Red arrows indicate phage lysis and lysogenic genes; blue arrows indicate phage structural genes (tail, capsid and fiber); green arrows indicate replication, recombination, repressor and phage related genes; gray arrows indicate hypothetical proteins. The black knobs indicate ρ-independent terminator and the bent arrows indicate putative promoters. The star indicates phage tRNA. **(c)** the attachment sites *attP* and *attB* of ɸ437. The *att* sites were analyzed using motif-finding tools MEME^49^. The attachment sites on the tRNA *P*. *terrae* BS437 (*attB*) and ɸ437 (*attP*) are shown.

TEM was then used to observe phage progeny in the MMC-induced lysates as well as controls, and to determine the morphology of the phage particles (Fig. 2a). First, phage particles were not observed in the controls, even after extensive screens. However, in the MMC-induced suspensions, homogeneous populations of virions were found. These particles were composed of isometric heads of ~50 nm in diameter, and contractile tails with base plates of about ~75 nm length. Two to three long tail fibers were also distinguishable. According to morphological classification criteria [ICTV - International committee on taxonomy of viruses], the phage can be classified as belonging to the *Myoviridae*, with typical contractile tail features.

Thus, high levels of MMC induced lysis of BS437 cells, albeit partially, which occurred concomitantly with the release of TEM-detectable phage particles (Fig. 2). We then tested the potential infectivity of the released phage particles using the DAL method and spot test with diverse indicator hosts (Table S1), including *P*. *terrae* BS437. In several attempts (adding different concentrations of helper salts MgCl_2_, MnCl_2_ and CaCl_2_), the phage lysates did not give rise to any plaque on the different hosts tested. We also examined whether any integration event had taken place on selected hosts, using suites of 20 host clones taken from the areas where lysates were spotted (Fig. S1). The clones were PCR-screened using phage ɸ437 major capsid specific primers (see Material and Methods). The results showed that any integration event that might have occurred was below the detection limit of the applied method.

Linking the phage particle population to prophage ɸ437 specific genes using qPCR. We estimated that the phage lysates, estimated to have raised number of phage particles per ml (about 10^8^ in the MMC-10 induction), contained dominant phage ɸ437 particles. To examine this tenet, we thus developed and performed phage ɸ437 based real-time quantitative PCR^34, 35^, on extracts prepared from the control and the MMC (4 µg/mL and 10 µg/mL) induced phage lysates.

The results confirmed that the phage ɸ437 progeny levels increased over time in correspondence with the MMC concentration, with the highest gene copy number being 7.60 × 10^8^ per ml at 20 h with MMC-10 induction (ANOVA significant *n* = 3, *P* < 0.05). On the other hand, in the control (no MMC induction), the copy numbers were consistently low, i.e. about 6.88 × 10^6^ per ml at 20 h (Fig. 1b). This result indicated that (1) phage ɸ437 - upon MMC induction - is indeed induced from the BS437 genome by MMC to form progeny, and (2) it most likely concurs with the phage particles visualized by TEM, as described in the foregoing. Furthermore, we found a consistent presence of about 10^6^ to 10^7^ copies of the gene for the phage ɸ437 major coat protein in the control, indicating spontaneous release of phage particles; as yet, we still do not understand what type of ‘cue’, e.g. partial/incidental stress, may have caused such release.

### Detailed analysis of the genome of phage ɸ437

The genome of phage ɸ437, as evidenced from virion population sequencing, was approximately 54 Kb in size, with GC-content of about 60.31%. This is slightly below the GC content of the host bacterium *P*. *terrae* BS437, of about 61.78%. Based on RAST annotation, the phage ɸ437 genome was found to contain 90 predicted open reading frames (ORFs), with 63 ORFs having more than 100 bp, 83 ORFs having start codon ATG (92%), four GTG (4%) and three TTG (3%). The identified PP region in BS437 (using our criteria, see materials and methods) was smaller than the sequenced genome of ɸ437. However, we did find that the PHAST-identified PP region had about 54 Kb in recent analysis. The comparison of the initially-identified smaller region with the sequenced ɸ437 genome is shown in Fig. S2.

The biggest predicted gene in the genome of phage ɸ437 was *orf88*, of 1,688 bp (563 amino acids - aa). The predicted gene product was identified as a portal protein, which enables DNA passage during ejection and virion assembly. The predicted protein had 35% homology [90% coverage] to a similar one from *Xylella* phage Sano (AHB12085). The smallest gene (*orf31*) had only 126 bp (41 aa), and the predicted protein had 61% homology [43% coverage] to a tail fiber protein of *Escherichia* phage 64795_ec1 (YP_009291518). Interestingly, more than half of the genes of the ɸ437 genome (53 genes, 59%) were predicted to encode hypothetical proteins (Table 1), with no designated phage sequences. This indicates the phage is indeed a repertoire of novel genes. To assign functions to the hypothetical gene products, PSI-BLASTP and Phyre^2^ were used (see Materials and Methods), as detailed in the following.

#### Predicted genes encoding proteins that determine phage lifestyle

Phage ɸ437 was predicted to have a predominantly temperate lifestyle in its natural setting, as first evidenced by the fact that it was detected as a complete prophage. This tenet was also supported by PHACTS-supported and genomic analyses that showed the presence of typical genes involved in lysogeny. First, the phage ɸ437 genome encodes a predicted integrase (*orf47*), with 33% homology [40% coverage] to *Pseudomonas* phage D3 integrase (NP_061531). This integrase belongs to the tyrosine recombinase family, and a typical family representative is the phage lambda integrase^36^. We also found a tRNA sequence in the intergenic region adjacent to the integrase-encoding gene (Fig. 2b,c). We predict this site to be the phage integration site^12, 36^. A second piece of evidence for the prophage lifestyle of ɸ437 was the presence of phage lambda-like repressor genes (*orf27*), next to an antirepressor (*orf65*), indicating the presence of a system designed to ‘hold’/’release’ the integrated form.

#### Tail component and DNA packaging genes

As shown in Table 1, 28 phage ɸ437 morphogenesis genes were found, i.e. *orf3*, *5*, *23*, *28*, *31*, *48*, *51*, *54*, *56*, *57*, *62*, *70–77*, *79*, 80, *82*, *84–88* and *90*. PSI-BLASTP analyses of these genes showed homologies with database entries at 24–61% similarity and at coverages of 16–100%. PSI-BLASTP and Phyre^2^ analyses revealed that some ORFs encoded hypothetical morphological proteins. Thus, predicted tail fiber protein (*orf56*) showed 45% homology [75% coverage] with a gene of *Salmonella* phage IME207 (YP_009322735). Phage ɸ437 also contained ORFs predicted to encode several baseplate proteins (*orf51*, *53*, *72 and 74*). Thus *orf51* and *orf72* may encode baseplate assembly proteins, as they showed 31% [26% coverage] and 32% homologies (89% coverage) with such ORFs from *Escherichia* phage Lw1 (YP_008060715) and *Shigella* phage SfIV (YP_009147467), respectively. Fifteen ORFs were predicted to encode a suite of tail proteins (*orf3*, *23*, *31*, *54*, *56*, *57*, *62*, *70*, *71*, *73*, *75*, *76*, *77*, *79* and *80*), next to a tail sheath protein (*orf80*). The latter showed 42% homology [100% coverage] to a putative tail protein in *Enterobacteria* phage SfI (YP_009147459). The products of *orf84* through *orf90*, next to *orf48* and *orf5*, were predicted to be involved in the packaging of DNA and in capsid formation (Table 1); the major capsid protein (*orf84*) showed 38% homology [99% coverage] to a similar protein from *Aurantimonas* phage AmM-1 (YP_009146944). The phage ɸ437 genome also contained a putative ORF encoding a portal protein (*orf40*) as well as an ORF for a large terminase subunit protein (*orf42*). These proteins showed 35% [90% coverage] and 28% homology [30% coverage] to their database counterparts, respectively. These proteins are all essential in phage DNA packaging processes.

### The phage ɸ437 genome - comparison to related sequences and phylogenetic tree

**Figure 3:**
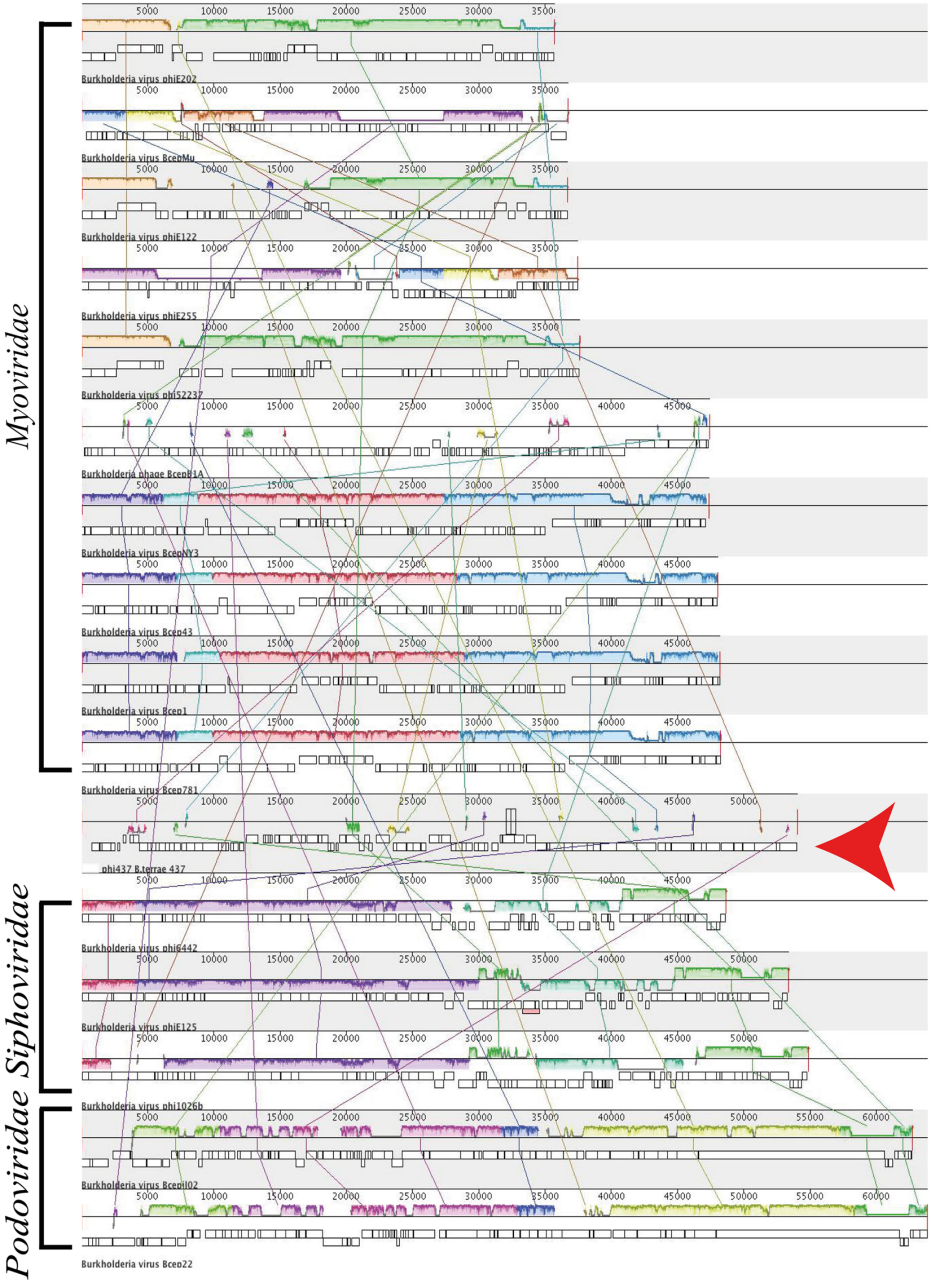
The Multiple genome alignment of *Burkholderia* phages. Genome were compare using progressiveMauve software, the genome homologous indicates by the local coliner blocks (LCB) and connected with lines. The analysis included known *Burkholderia* phages from *Myoviridae*, *Siphoviridae* and *Podoviridae*. The ɸ437 is indicated by red arrow.

**Figure 4:**
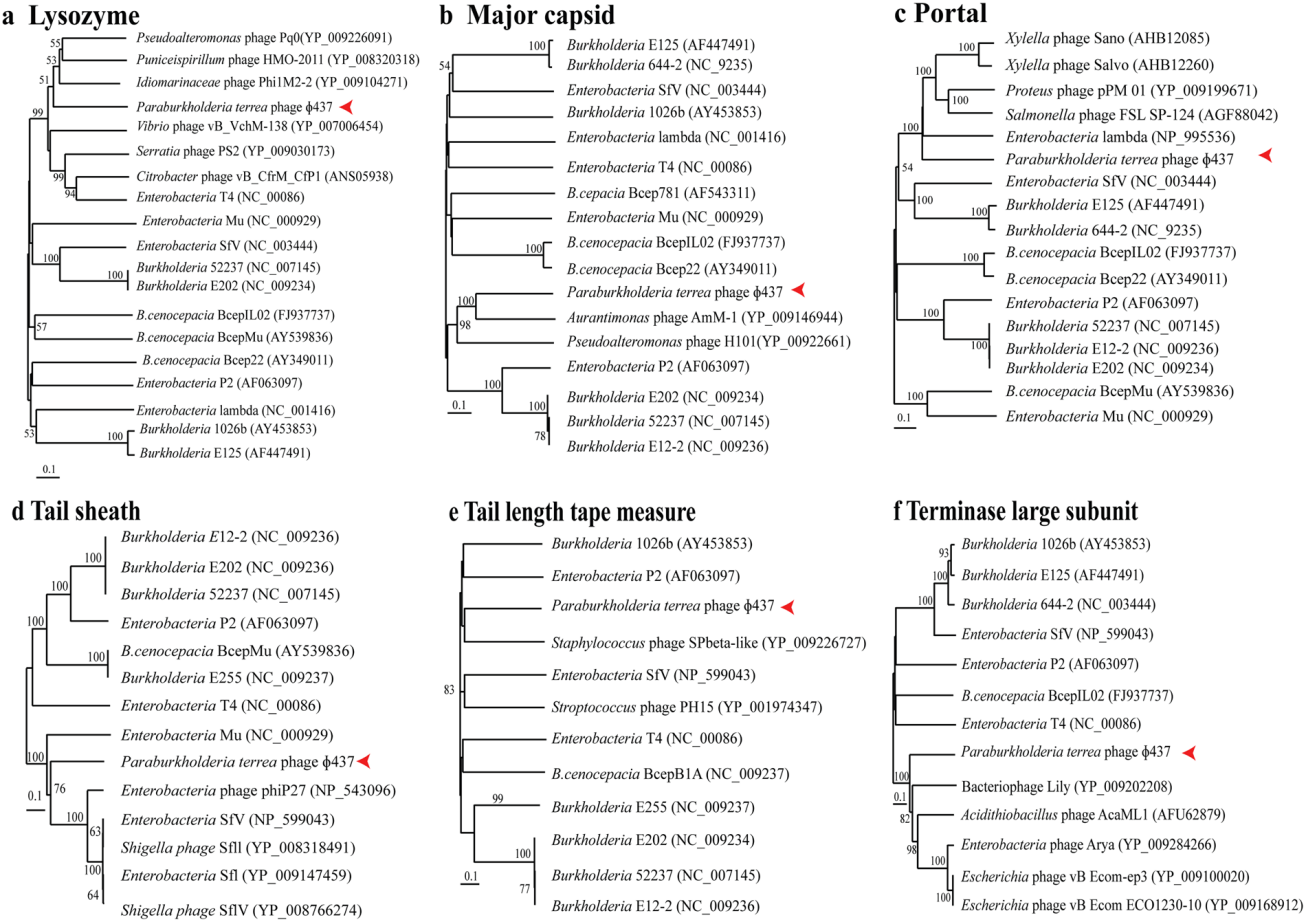
Comparison of (**a**) *Burkholderia* virus E125 (AF447491), (**b**) *Paraburkholderia terrae* phage ɸ437,and (**c**) *B. pseudomallei* phage 1026b (AY453853). Color boxes are indicated as previous figure with additional. Comparison percentage was generated using BLAST + 2.4.0 (tBLASTx with cutoff value 10^−3^) and map comparison figures were created with Easyfig as indicated in material and methods. Gene similarity percentage is indicated in gray scale bar.

In this analysis, a holistic approach was used, in which phylogenetic and overall DNA and protein sequence identities were used as the criteria. First, BLASTN analyses of the ɸ437 genome showed no similarity of the whole sequence to sequences present in the viral (tailed-phage) database. Subsequent PSI-BLASTP analyses revealed that proteins encoded by 19 of the 90 genes of the ɸ437 genome showed best hits to proteins encoded by other *Burkholderia* phages (Table 1). We thus compared the ɸ437 genome sequence to those of known *Burkholderia* phages (see Materials and Methods) using progressiveMauve^37^, pairwise comparisons and nucleotide dot-plot analyses. The progressiveMauve analyses showed non-colinear synteny of the *P*. *terrae* phage ɸ437 sequence with those of other *Burkholderia* phages (Fig. 3). Then, pairwise comparisons of the phage ɸ437 sequence to those of *Burkholderia* virus E125 (AF447491) and *B*. *pseudomallei* 1026b (AY453853) [both with similar genome sizes, i.e. 53.4 Kb and 54.8 Kb] confirmed the similarity, at a very low level, of phage ɸ437 with other *Burkholderia* phages (Fig. 4). Finally, the dot-plot analyses also showed low similarities among the compared sequences (Fig. S3). Collectively, these results supporting the BLASTN and PSI-BLASTP analyses.

**Figure 5:**
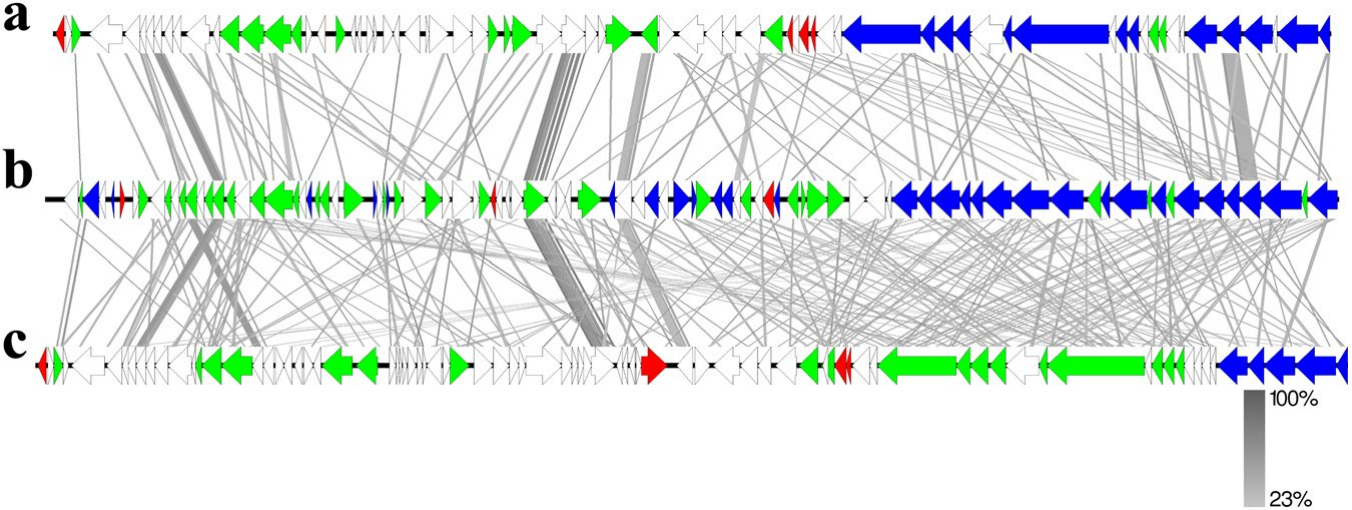
Phylogenetic trees of phage ɸ437 for (**a**) lysozyme, (**b**) major capsid, (**c**) portal, (**d**) tail sheath, (**e**) tail length tape measure and (**f**) phage terminase large subunit. Phylogenetic tree were generated with neighbor-joining tree Mega version 7 with 1,000 boothstrap method and *p*-distance methods. Red arrows indicate ɸ437. PSI-BLASTP best hits, coupled with other known *Burkholderia* phages were used in the analysis.

Phylogenetic analyses were then performed on the basis of selected proteins encoded by ɸ437, using MEGA7 [see Materials and Methods]. We thus analyzed phage hallmark genes, i.e. those encoding (1) lysozyme, (2) the major head capsid protein, (3) the portal, (4) the tail sheath protein, (5) the tail length tape measure protein and (6) the phage terminase large subunit. The closest hits to these proteins were most often proteins predicted from other phages (Fig. 5). The trees thus consistently pointed to a relatedness of ɸ437 to other phages. However, the phage ɸ437 proteins were phylogenetically quite distantly related to similar proteins from other phages. Specifically, the phage ɸ437 encoded 150-aa lysozyme had 40% homology [94% coverage] to similar proteins encoded by *Idiomarinaceae* phage Phi1M2-2 (YP_009104271), classified to the family *Siphoviridae*. Moreover, the 354-aa major capsid protein showed 38% homology [99% coverage] to a similar protein encoded by *Aurantimonas* sp. phage AmM-1 (YP_009146944), which was classified to the family *Caudoviridae*. The 562-aa portal protein had 35% homology [90% coverage] to a similar protein encoded by *Xylella* phage Sano (AHB12085), classified to family *Siphoviridae*. The 496-aa tail sheath protein had 42% homology [100% coverage] to a tail sheath protein from *Enterobacteria* phage SfI (YP_009147459), classified to the family *Myoviridae*. The 520-aa tail length tape measure had 28% homology [28% coverage] to a similar protein from *Burkholderia* phage BcepB1A (YP_291174), classified to the family *Myoviridae*. Finally, the 416-aa phage terminase large subunit had 44% homology [81% coverage] to a similar protein from *Acidithiobacillus* phage AcaML1 (AFU62879), classified to the family *Myoviridae*. These results show an overall consistent yet low level of similarity to proteins from known phages, indicating (1) phage ɸ437 predicted proteins are related to similar ones from phages, and (2) overall, phage ɸ437 is only remotely related to any known phage.

### Phage core genes versus predicted morons

Given the large genetic distance of most ɸ437 genes to genes of known phages (Fig. 3), it was difficult to identify morons in the ɸ437 genome sequence. However, some genes with features that were strongly suggestive of morons were found (Fig. 2b). In this study, we applied strict criteria for protein-encoding regions to be considered to constitute a moron: (1) they potentially give fitness advantages to the host and do not constitute phage core genes, (2) they are flanked by an upstream σ^70^ promoter and a downstream *ρ*-independent transcriptional terminator, allowing autonomous transcription^38^. Genes meeting criterion (2) were found in several putative intergenic regions (Fig. 2b). As a third criterion (criterion 3), we used the fact that morons often have GC-contents different from those of neighboring sequences^38^. Thus, *orf64* was singled out as a potential moron; the region was identified as a so-called *amrZ* (alginate and motility regulator Z)/Arc domain. PSI-BLASTP analysis showed *orf64* has 55% homology [79% coverage] with a similar protein present in *Pseudomonas* phage SM1 (ALT58107). This result was supported by Phyre^2^ analysis (Table S3). Furthermore, 55% homology [80% coverage] - with 100% confidence – was found with ‘alginate and motility regulator Z’ found in *Pseudomonas aeruginosa*. The *orf64* encoding transcriptional factor *AmrZ* was homologous to the *Pseudomonas* phage SM1 (ALT58107) Arc domain which had been shown to regulate virulence during infection^39^. This factor is also essential for biofilm formation in *Pseudomonas aeruginosa*.

## Discussion

In spite of the apparent selection and outgrowth in mycosphere soils of the *Paraburkholderia* types used as phage hosts, to our surprise we could not detect any phage that was productive (including highly lytic to temperate modes of action) on these. This indicated that such phage populations, if present, were very low in number, so that they were not detectable by the classical DAL or related spot tests. Alternatively, our indicator bacteria (Table S1) may have had effective defense systems against the extant phage populations, which may have included R-M, CRISPR or BREX systems^1, 40^. Finally, the conditions that allow such phages to proliferate on DAL plates may not have been established in our screens. We thus set out to analyze the genomes of several selected mycosphere-isolated *Paraburkholderia* strains for predicted prophage sequences using currently accepted bioinformatics tools.

The analysis of the genomes of our *Paraburkholderia* strains to identify prophages/phage-like elements (PP) showed evidence for the contention that all of the analyzed sequences contain substantial amounts of prophage regions. Most of the identified PP regions turned out to be remnants of a phage ‘history’, as previously discussed^12, 13^. These regions have probably been subjected to (stochastically acting) selective deletion pressures from the host cell, which may indicate their infrequent (re)selection. When phage structural machinery genes get eroded, prophages lose their abilities to produce progeny. Such prophages might still be coding and remain functional as they offer lysogenic conversion to host cell^11^ or they increasingly might represent ‘passive genetic cargo’ that is not transcribed^12^. With respect to the identified phages, such hypotheses surely need experimental evidence.

A certain prevalence of prophages in the *Paraburkholderia* genomes was expected considering the fact that these *Paraburkholderia* species can inhabit the mycosphere, an environment that has been depicted as a hot spot for HGT processes in soil^27^. So far, only few studies have successfully described phages from *Burkholderia* (and/or *Paraburkholderia*) spp.^41–45^. However, most phages described were from pathogenic strains isolated from clinical environments, i.e. *B*. *cepacia* complex isolates. To the best of our knowledge, no previous studies have as yet focused on *Paraburkholderia* phages in environmental isolates, especially from the mycosphere. We here singled out the *P*. *terrae* strain BS437 phage ɸ437, on the basis of the experimental and computational analysis, as outlined in the foregoing.

Phage ɸ437 was apparently ‘spontaneously’ released in strain BS437 populations growing in liquid medium, whereas its particle numbers were raised by successful induction with MMC (Fig. 1a). These observations were supported by the concomitant phage coat gene based qPCR analyses and TEM observations (Figs 1b and 2a). However, we did not detect any infective phage particles by the DAL or spot tests applied to phage lysates, which may be due to (1) the absence of infectivity in our phage lysate, or (2) an intrinsic resistance or insusceptibility of host cells to released phages, as previously observed in other study. Notably, 45 strains of *Clostridium difficile* also failed to show infective phage production using the DAL method^9^. The isolation, propagation and downstream analysis of phages from natural samples remain a challenge^46^. The absence of detectable phage activity in the spot tests clearly excluded a lysis-from-without scenario under these conditions.

The spontaneous prophage induction that was observed in the liquid controls used [non-MMC induction] (Fig. 1a), if occurring in natural settings, might have an impact on host fitness^10^. We hypothesized that ɸ437 might modulate the formation of *P*. *terrae* BS437 biofilms on its fungal host strain, which we presume to be akin to *P*. *terrae* strain BS001 forming biofilms on *Lyophyllum* sp. strain Karsten^47^. However, experimental work still needs to be done to prove this theory. Collectively, the significant decrease of the OD_600_ in strain BS437 cultures upon MMC induction, the phage progeny observed by TEM, and the increased gene copy number of the ɸ437 major capsid gene strongly indicate that phage ɸ437 was the major, if not only, phage that was released from the genome of *P*. *terrae* BS437.

The genomic architecture of ɸ437, compared to *Burkholderia* virus E125 (AF447491) and *B*. *pseudomallei* 1026b (AY453853) indicated a strong conservation of a cluster of functional genes (phage core genes) in the same relative spatial position. Tail (*orf70-orf80*) and head (*orf84-orf90*) morphogenesis genes were among the most conserved genes in the ɸ437 genome. This is consistent with data by Morgan *et al*.^48^ and Summer *et al*.^41^, indicating that such conserved genes as well as gene order represents a phage gene repertoire that is fine-tuned to effectively execute key phage functions (as shaped by evolution). Moreover, the key functional genes may be better interchanged in the continuous flux of gene acquisition and recombination in the bacterial host genome. The analyses applied to assign the taxonomic class of ɸ437 show no large sequence similarity to any known phage sequences in the public database. However, the phylogenetic analysis of the selected phage hallmark genes (phage lysozyme, major capsid, portal, tail sheath, tail length tape measure and phage large terminase subunit) revealed ɸ437 to be most related to phages from the *Myoviridae* family. Moreover, the morphology of ɸ437 placed it in the *Myoviridae*. We thus propose ɸ437 as a new member of this family, with unique sequence features that do not relate to any of the currently ICTV-recognized subfamilies or genera.

The integration of phage ɸ437 is not well understood and does not fit classical integration mechanisms. We found the site/region of integration in the host bacterium and phage genome showed interrupted blocks, regardless of sequence identity. It is noteworthy that comparative studies of lambdoid bacteriophage genomes^11^ also revealed mosaicisms as a consequence of HGTs involving homologous and non-homologous recombinations^49, 50^. Additionally, moron genes have been reported to be common in *Burkholderia* phages^44^. Our analyses found one moron (*orf64*) that potentially endows the host with a superinfection defense mechanism against other phage infection, enhance host fitness and enhance biofilm formation. Considering this line of evidence, we hypothesize that the gene product potentially plays a role in the *P*. *terrae* strain BS437 interaction with a host fungus in the mycosphere, including biofilm formation. Although the significance of this potential moron still remains enigmatic at this point, this analysis gives direction for future experiments.

## Materials and Methods

### Phage isolation from soil and mycosphere samples

Replicate soil and mycosphere samples (*Scleroderma citrinum* and *Galerina* spp.) were obtained from a forest in Noordlaren in autumn 2015, and processed as in Zhang *et al*.^27^. Attempts to isolate phage from these samples were made using two methods. First, 0.5 g of each mycosphere sample was added to 5 ml of sterile water, after which the mixtures were vortexed vigorously. After one minute still, centrifugation at 100 *xg* (30 s) was done to sediment course soil particles. The collected supernatant was then spun at maximal speed (7,000 *xg*) for 15 min, to remove fine soil particles. Following this, 100 µL was filtered over Whatman 0.22 µm cellulose acetate filter (GE Healthcare Life Sciences, Pittsburgh, PA, USA); the suspension was then added to 20 mL of LB (Sigma-Aldrich, St. Louis, Mo, USA), with 200 µL of overnight grown ‘indicator’ bacteria (Table S1). The suspensions were incubated overnight at 28 °C.

Method 2 consisted of directly adding 0.5 g soil or mycosphere sample to 20 mL LB broth and incubating overnight at 28 °C, to foster bacterial growth and potential phage development. Following incubation, the cultures were centrifuged at maximal speed (7,000 *xg*) for 10 min at 4 °C to pellet bacterial cells, and supernatants filtered over Whatman 0.22 µm cellulose acetate filter (GE Healthcare Life Sciences, Pittsburgh, PA, USA). One mL of each filtered supernatant was then added to 3 mL indicator bacteria (Table S1) in LB medium, and incubated overnight at 28 °C. The resulting cultures were then centrifuged at maximum speed for 30 min at 4 °C and the filtered supernatants used for later cultures. The procedure was repeated five times, ultimately yielding a suspension that presumably contains phage particles^51^.

### Prophage identifications across genomes

The genomes of the selected *Paraburkholderia* strains were screened for the presence of prophages by using PHAST^31^- version October 2015, Prophinder/ACLAME^32^- version 04, October 2015 and PhiSpy [PhiSpyNov11_3.2]^33^. PHAST and Prophinder identify prophage regions by using a database of known phage genes, sequence identification, tRNA identification (as phages often use tRNAs as target sites for integration), attachment site recognition and gene clustering density measurements (prophage regions can be identified as clusters of phage-like genes within a bacterial genome)^31, 32^. PhiSpy uses several distinct characteristics of prophages, as outlined in the following. First, the median length of predicted proteins; as the median protein lengths in phage regions is much higher than that of proteins in the bacterial genome. Additionally, the directionality of the transcription strand and the GC skew. Both directionality of the transcription strands and GC skew are correlated with the direction of replication. Most consecutive genes in phage genome tend to be encoded on the same strand, in contrast to bacterial consecutive genes. Any observed changes in GC skew might result from the insertion of foreign DNA. Also, the abundance of unique phage words is used, next to the phage insertion site (*attP*) and the similarity to known phage proteins^33^. We here also applied other criteria to define putative prophage-like (PP) regions: (1) PP of sizes below 10 Kb were discarded^5, 11^ and (2) when a region consistently appeared in all three independent analyses, we used the PHAST results, as PhiSpy was reported to give less consistent results^52^.

### Bacterial growth and MMC-mediated prophage induction

*Paraburkholderia terrae* strain BS437 became the focus of this study. It was isolated from the mycosphere of *Lyophyllum* sp strain Karsten^28^ and is a current reference strain in our laboratory. The strain was grown in LB broth at 28 °C with shaking (180 *rpm*). Induction with MMC (Sigma-Aldrich, St. Louis, Mo, USA) was conducted according to Fortier and Moineau^9^, with modifications. Briefly, bacterial cells were introduced into 5 ml of LB medium and incubated overnight at 28 °C (shaking at 180 *rpm*). The resulting cultures were then transferred (1:100) into replicate Erlenmeyer flasks containing 40 ml of fresh LB medium and growth was monitored until the exponential growth phase (about 10 h incubation). Thereafter, all cultures were split into two 20 ml cultures. MMC was added to the cultures, at final concentrations of either 4 or 10 µg/mL (MMC-4, MMC-10, respectively), with the ‘twin’ culture serving as the control. The cultures were incubated and the OD_600_ was monitored for 24 h. Decreases of the cell density were taken as indications of progressive cell lysis and prophage release. The experiments were done with three biological replicates. The resulting crude lysates were finally filtered over Whatman 0.22 µm cellulose acetate filter (GE Healthcare Life Sciences, Pittsburgh, PA, USA) and stored at −20 °C until further analysis.

### Assessment of host range and indicator bacterial strains

For all phage activity tests, the double agar layer (DAL) method, next to a spot test, was used according to Adams^30^, with some modifications. In one effort, we used the extracted mycosphere and bulk soil directly with selected indicator *Paraburkholderia* strains (Table S1). Suspensions resulting from the fivefold enrichment with the same indicator bacteria were also used.

Spot or “lysis from without” assays were also used on the induced lysates. Briefly, overnight cultures of the indicator bacteria (Table S1) were poured onto R2A (Becton Dickinson, NJ, USA) plate agar. Then 5 µL (10^−2^, 10^−3^, 10^−4^, 10^−5^) diluted induced lysates were spotted onto the plate and the plates incubated overnight at 28 °C.

### Quantitative PCR (qPCR)

Specific primer sets for detecting phage genes were developed as the indicator gene to verify the presence of phage ɸ437 in the induced lysate. We selected one phage ɸ437-specific gene: a major capsid protein using the *P*. *terrae* BS437 draft sequence^26^. Major capsid genes have been used to assess viral diversity (see review by Adriaenssens and Cowan^53^). This method followed the path taken to quantify ten closely related lambdoid phages of *Escherichia coli* strain K-12^34, 35^.

Here, we treated the induced lysates and the control (not treated with MMC) with DNase to remove any host genomic DNA (confirmed by host-specific PCR). Using the ɸ437 specific primer set, a 198 bp band was produced from *P*. *terrae* BS437 DNA, whereas no bands were amplified from genomic DNA of *P*. *terrae* strains BS001, BS007, BS110, 17804^T^ or *P*. *hospita* DSMZ 17164^T^ and *P*. *caribensis* DSMZ 1323^T^ (Fig. S1a). Then, these strains were used to detect and quantify phage progeny in the induced lysates as described^34, 35^.

Briefly, induced cultures were centrifuged and filtered over Whatman 0.22 µm puradisc syringe cellulose acetate filters (GE Healthcare Life Sciences, Pittsburgh, PA, USA) to remove bacterial cells and debris. A drop of chloroform was added to 10-fold diluted filtrates. These were then centrifuged at 2700 *xg* for 10 min at 4 °C. Then, 2 units of DNaseI endonuclease (Sigma-Aldrich, St. Louis, Mo, USA) with 1.3 µL 10x reaction buffer (Sigma-Aldrich, St. Louis, Mo, USA) was added to 10 µL lysate and the mixture was kept at 37 °C for 1 h. Later 1.5 µL of stop solution (Sigma-Aldrich, St. Louis, Mo, USA) was added and the mixture incubated at 95 °C for 30 min to inactivate DNaseI and also to open up phage capsids. The resulting suspensions were then diluted 10 fold and stored at −20 °C for later analysis. Primers specific for the ɸ437 gene for major capsid protein were used (PP1.437_ca1F: 5′-CACGATGACACGATCCACAC-3′; PP1.437_ca1R: 5′-GAGAACCATGCCCTGAACC-3′). The qPCR reaction mixtures consisted of 12.5 µL SYBR Green (Applied Biosystems, CA, USA), 0.75 µL each primer (Eurogentec, Liège, Belgium), 10 µL ultrapure water and 1 µL sample, for a total 25 µL reaction volume. Amplification and detection of ɸ437 product were performed using ABI 7300 (ThermoFisher Scientific, Waltham, Mass, USA) with qPCR reaction conditions: denaturation at 95 °C for 30 sec, annealing at 60 °C for 1 min and elongation at 72 °C for 60 sec. The qPCR efficiency was 106%.

### The examination of the presence of prophage within indicator hosts

Experiments were performed to test the potential integration of ɸ437 (Fig. S1) using spot tests with ɸ437 containing suspensions (titer estimated at 10^8^ per ml) on several *Paraburkholderia* strains (*P*. *terrae BS001*, *BS007*, *BS110*, 17804^T^, *P*. *hospita* DSMZ 17164^T^ and *P*. *caribensis* DSMZ 1323^T^) as previously explained. The top and bottom parts of each spots were later streaked onto the new R2A medium and incubated overnight at 28 °C. Colony PCR-based test using specific ɸ437 gene for major capsid protein (198 bp) were used and 20 single-colonies from each strains were tested. The isolated DNA of ɸ437 and the phage suspension produced from strain BS437 were used as positive controls, whereas the unspotted strains and *E*. *coli* K-12 were used as negative controls. The test was applied to potential host strain BS007, with 50 more single-colonies.

### Phage particle concentration by polyethylene glycol (PEG) 8000

The induced phage particles were purified according to the PEG method of Sambrook and Russell^54^ with the following modifications. Induced phage lysate was centrifuged at 11,000 *xg* for 15 min at 4 °C, and then supernatants were filtered over a Whatman 0.22 µm puradisc syringe filter- cellulose acetate (GE Healthcare Life Sciences, Pittsburgh, PA, USA). NaCl (29.2 g) was dissolved into 500 mL lysates to final concentration 1 M, which was then stored on ice for 1 h. Solid polyethylene glycol (PEG) 8000 was added to the supernatant to a final concentration of 10% (w/v) and the mixture stored overnight at 4 °C to allow phage particles to precipitate. The PEG-precipitated lysate was then centrifuged at 11,000 *xg* for 10 min at 4 °C (Sorvall SLA-1500 rotor). The supernatants were discarded to 20 mL and 10x SM buffer (10 mM NaCl, 50 mM Tris, 10 mM MgSO_4_, and 0.1% gelatin) was added for storage and later analysis.

### Phage DNA extraction and sequencing

Phage DNA extraction was performed with a Phage DNA Isolation Kit (Norgen, Biotek Corp, ON, Canada) using manufacturer’s protocols, with slight modification, i.e. DNase I inactivation temperature was 80 °C for 10 min. In addition, 16S rRNA PCR amplification using 16SFP/16SRP universal 16S rRNA gene primer set^55^ was performed to confirm the absence of genomic DNA in the phage DNA extracts. Aliquots of amplification products were electrophoresed in 1% agarose gels stained with ethidium bromide and visualized under UV illumination.

Phage DNA was sequenced on the Illumina HiSeq. 2500 paired-end by BaseClear (Leiden, Netherlands). The libraries for the strains were prepared using Illumina genomic Nextera XT libraries. The quality analyses of FASTQ sequence reads were done using the Illumina Casava pipeline version 1.8.3. The Initial quality assessment was based on data passing the Illumina Chastity filtering. Subsequently, reads containing PhiX control signal were removed using an in-house filtering protocol. In addition, reads containing (partial) adapters were clipped (up to minimum read length of 50 bp). The second quality assessment was based on the remaining reads using the FASTQC quality control tool version 0.10.0. The final quality scores per sample yielded 707,8049 reads, or 166 MB, at 37.45 average quality. Reads were then aligned and successfully assembled using the CLC genomics workbench 9 (Aarhus, Denmark) with the default parameters: mismatch cost 2, insertion cost 3, deletion cost 3, length fraction 0.5 and similarity 0.9.

RAST (Rapid Annotation using Subsystem Technology) was subsequently used to annotate the sequenced genome^56^. Predicted hypothetical proteins were checked with PSI-BLASTP and Phyre^2^ program^57^. Predicted amino acid sequences of genes with assigned function [and of those without] were analyzed against the non-redundant (nr) NCBI database and the tailed phages database by PSI-BLASTP. Phyre^2^ was used to predict secondary and tertiary structures (Table S2). To predict the lifestyle, PHACTS (uses a novel similarity algorithm to create a training set from known phage lifestyles and a random forest that classify a multitude of decision trees^58^) was used. Phage-bound σ^70^ promoters were predicted using predicted promoter tool (http://www.fruitfly.org/seq_tools/promoter.html) and *ρ*-independent terminators were identified using the Arnold terminator-finding program^59^. The analysis of tRNA in the phage genome was done using tRNAscan-SE^60^. The attachment (*att*) sites were analyzed using motif-finding tools MEME^61^. The PROBIUS prediction tool^62^ was used to predict transmembrane and signal peptide of genome ɸ437.

### Transmission electron microscopy (TEM)

Viral particles were detected, and viral morphology examined by TEM (PHILIPS CM10). The phage stocks were directly applied onto carbon-coated nitrocellulose grids, and let it set for about a minute. The excess of liquid was drained with filter paper before negative staining with 1% uranyl acetate followed by washing and drying, before immediate observation in the TEM.

### Genome comparison and phylogenetic trees

Known *Burkholderia* phages such as, *Burkholderia cepacia* phage Bcep22 (AY349011), *B*. *cenocepacia* phage BcepM(AY539836), *B*. *cenocepacia* phage BcepB1A (NC_005886), *B*. *pseudomallei* phage 1026b (AY453853), *Burkholderia* virus E125 (AF447491), *Burkholderia* phage BcepIL02 (FJ937737), *Burkholderia* phage 52237 (NC_007145), *Burkholderia* phage E202 (NC_009234), *Burkholderia* phage E255 (NC_009237), *Burkholderia* phage 644-2 (NC_009235), *Burkholderia* phage E12-2 (NC_009236), *Burkholderia* phage Bcep1 (NC_005263), *Burkholderia* phage Bcep43 (NC_005342), *Burkholderia* phage Bcep781 (NC_004333) and *Burkholderia* phage BcepNY4 (0096001), including *Enterobacteria* phage T4 (NC_00086), *Enterobacteria* phage Mu (NC_000929), *Enterobacteria* phage sfV (NC_003444), *Enterobacteria* phage P2 (AF063097), and *Enterobacteria* phage lambda (NC_001416), coupled with the PSI-BLASTP best hits for hallmark genes (phage lysozyme, major capsid, portal, tail sheath, tail length tape measure and phage terminase large subunit gene) were used to generated phylogenetic trees and molecular evolutionary analysis. Trees were analyzed using MEGA7^63^. The comparison were performed with three different approaches, such as ProgressiveMauve^37^, pairwise comparison^64^ and dot-plot analysis^63^. Pairwise analysis generated by BLAST + 2.4.0 (tBLASTx with cutoff value 10^−3^) and map comparison figures were created with Easyfig.^64^. Dot-plot analysis was done using Gepard with default parameters^65^.

## Electronic supplementary material

Supplementary information
