# Supplementary material for: A novel inducible prophage from the mycosphere inhabitant Paraburkholderia terrae BS437
Source: Sci Rep. 2017 Aug 22;7:9156. doi: 10.1038/s41598-017-09317-8 (PMC5567305; doi:10.1038/s41598-017-09317-8)
Supplement: Supplementary file 1 — Supplementary information [file 41598_2017_9317_MOESM1_ESM.docx]

**Supplementary information**

**A novel inducible prophage from the mycosphere inhabitant *Paraburkholderia terrae*  BS437**

Akbar Adjie Pratama^1*^, Jan Dirk van Elsas^1*^

^1^Department of Microbial Ecology, Microbial Ecology - Groningen Institute for Evolutionary Life Sciences, University of Groningen, Nijenborgh 7, Groningen, 9747 AG, The Netherlands.

*Correspondence and requests for materials should be addressed to A.A.P. (email: a.a.p.pratama@rug.nl) or J.D.V.E. (email: j.d.van.elsas@rug.nl).

**Table S1 *Paraburkholderia* strain used in double agar layer method (DAL) and spot-test for screening and host-range test.**

| **Bacteria** | **Strains** | **DAL^a^** | **Spot-test^a^** |
| --- | --- | --- | --- |
| *Paraburkholderia terrae* | BS001 | N | N |
|  | BS007 | N | N |
|  | BS110 | N | N |
|  | BS437 | N | N |
|  | DMSZ 17804T | N | N |
| *P.phytofirmans* | BS455 | N | N |
|  | BIFAS53 | N | N |
|  | JU15 | N | N |
|  | PsJN | N | N |
|  | BS413 | N | N |
|  | BS410 | N | N |
|  | BS420 | N | N |
|  | BS421 | N | N |
|  | BS425 | N | N |
| *P.caribensis* | DSMZ 1323T | N | N |
| *P.hospita* | DSMZ 17164T | N | N |
| *P.tericolla* | BS430 | N | N |
|  | BS454 | N | N |

**^a^**DAL: Double agar layer method; N: no plaque forming observed

**Table S2. General result of putative prophage regions analysis across *Paraburkholderia* genomes**.

| **Bacteria** | **Strain** | **Acc. No^a^** | **Reference** | **Bacteria genome (bp)** | **No. of PP^b^** | **Total PP regions genome (bp)** | **% PP^b^** |
| --- | --- | --- | --- | --- | --- | --- | --- |
| *P.terrae* | BS001 | This study | 22, 24 | 11294072 | 17 | 923462 | 8,2 |
|  | BS007 | This study | 26 | 11024679 | 23 | 1299512 | 11,8 |
|  | BS110 | This study | 87 | 11176041 | 17 | 1114486 | 9,9 |
|  | BS437 | This study | 26 | 11301206 | 25 | 1274709 | 11,3 |
| *P.phytofirmans* | BS455 | This study | 26 | 8859905 | 8 | 390292 | 4,4 |
|  | BIFAS53 | This study | 88 | 8267758 | 8 | 224069 | 2,7 |
|  | J1U5 | This study | 88 | 10330795 | 27 | 1387966 | 13,4 |
|  | PsJN | NC_010681 | 89 | 4467537 | 2 | 78829 | 1,8 |

^a^Acc.No: accession number; ^b^ PP: putative prophage-like elements.

**Table S3. Phyre^2^ analysis of genome ɸ437^a^.**

| **ORF** | **aa length** | **GC%** | **kDa** | **pI** | **PDB molecule** | **Con (%)** | **Cov (%)** | **Id (%)** | **Motif(TMD, SP)** |
| --- | --- | --- | --- | --- | --- | --- | --- | --- | --- |
| 1 | 197 | 60.1 | 21,67 | 6.31 | Putative DNA-binding domain | 97 | 29 | 21 | N/N |
| 2 | 47 | 59.03 | 5,17 | 11.79 | LuxS/MPP-like metallohydrolase | 17 | 85 | 23 | N/N |
| 3 | 227 | 60.08 | 24,97 | 10.14 | Alpha-catenin/vinculin | 72 | 21 | 26 | N/N |
| 4 | 90 | 62.27 | 9,9 | 8.06 | Putative nucleoprotein | 16 | 9 | 75 | N/N |
| 5 | 41 | 53.17 | 4,51 | 4.08 | Inhibitory polypeptide | 11 | 15 | 83 | N/Y |
| 6 | 69 | 50.47 | 7,59 | 7.81 | Cell wall binding repeat | 12 | 16 | 27 | N/Y |
| 7 | 83 | 55.16 | 9,13 | 9.10 | Coil-vald | 61 | 31 | 46 | N/Y |
| 8 | 139 | 49.05 | 15,29 | 4.94 | Voltage-gated potassium channels | 84 | 23 | 34 | Y/N |
| 9 | 202 | 58.29 | 22,22 | 5.50 | Homeodomain-like | 39 | 7 | 27 | N/N |
| 10 | 84 | 65.09 | 9,24 | 10.02 | Binding protein glucocorticoid receptor dna-binding factor 1 | 62 | 36 | 20 | N/N |
| 11 | 117 | 54.23 | 12,87 | 5.83 | Hydrolase | 21 | 37 | 19 | N/N |
| 12 | 87 | 61.36 | 9,57 | 5.66 | HAD-like | 59 | 28 | 21 | N/N |
| 13 | 157 | 62.02 | 17,27 | 8.95 | Acyl-CoA N-acyltransferases (Nat) | 97 | 74 | 15 | N/N |
| 14 | 105 | 60.37 | 11,55 | 9.29 | Immunoglobulin-like beta-sandwich | 38 | 24 | 20 | N/N |
| 15 | 111 | 60.11 | 12,21 | 5.75 | Uncharacterized protein drra | 45 | 20 | 32 | N/N |
| 16 | 161 | 62.34 | 17,71 | 9.79 | Prophage-derived uncharacterized protein ybco | 100 | 47 | 28 | N/N |
| 17 | 120 | 62.80 | 13,2 | 4.56 | CMP/hydroxymethyl cmp hydrolase | 100 | 98 | 17 | N/N |
| 18 | 216 | 61.29 | 23,76 | 10.94 | Pre-mrna-splicing factor snu114 | 100 | 44 | 21 | N/N |
| 19 | 200 | 63.18 | 22 | 9.16 | Holliday junction resolvase RusA | 100 | 57 | 30 | N/N |
| 20 | 394 | 60.16 | 43,34 | 8.87 | DNA replication protein dnad | 99 | 22 | 15 | N/N |
| 21 | 184 | 61.17 | 20,24 | 9.41 | - | - | - | - | N/N |
| 22 | 103 | 63.78 | 11,33 | 9.89 | DNA-binding domain | 92 | 42 | 33 | N/N |
| 23 | 70 | 63.38 | 7,7 | 11.53 | Signaling protein t-lymphoma invasion and metastasis-inducing | 10 | 10 | 57 | N/Y |
| 24 | 63 | 52.60 | 6,93 | 5.52 | PABC (PABP) domain | 24 | 41 | 19 | N/N |
| 25 | 131 | 61.61 | 14,41 | 9.29 | Virulence-associated protein I | 95 | 50 | 15 | N/N |
| 26 | 95 | 60.76 | 10,45 | 9 | Transcription cro protein | 96 | 34 | 47 | N/N |
| 27 | 286 | 57.14 | 31,46 | 5.64 | Lambda repressor | 100 | 75 | 20 | N/N |
| 28 | 51 | 61.53 | 5,61 | 7.83 | Surfactant-associated protein d | 12 | 62 | 30 | N/N |
| 29 | 83 | 57.53 | 9,13 | 10.55 | 40s ribosomal protein s10, putative | 26 | 49 | 27 | N/N |
| 30 | 49 | 64.66 | 5,39 | 7.82 | Motor protein,protein transport myosin-vi | 21 | 27 | 54 | Y/N |
| 31 | 41 | 64.28 | 4,51 | 7.85 | Single transmembrane helix | 11 | 54 | 27 | N/Y |
| 32 | 110 | 62.16 | 62.16 | 5.86 | Signal recognition particle alu RNA binding heterodimer, SRP9/14 | 24 | 25 | 25 | N/N |
| 33 | 277 | 61.39 | 61.39 | 5.25 | Viral protein | 59 | 31 | 18 | N/N |
| 34 | 236 | 63.71 | 25,96 | 5.56 | PUA domain-like | 96 | 27 | 20 | N/N |
| 35 | 111 | 61.01 | 12,21 | 3.21 | Hydrolase, alpha-xylosidase bogh31a | 27 | 33 | 24 | N/N |
| 36 | 283 | 68.19 | 31,13 | 9.32 | Lambda repressor-like DNA-binding domains | 27 | 7 | 29 | Y/N |
| 37 | 84 | 60 | 9,24 | 6.16 | Activating signal cointegrator, RNA binding protein | 100 | 90 | 37 | N/N |
| 38 | 158 | 57.86 | 17,38 | 4.83 | Sulfite reductase [ferredoxin], chloroplastic | 25 | 29 | 20 | N/N |
| 39 | 64 | 55.89 | 7,04 | 4.45 | Transferase effector protein hopab2 | 30 | 30 | 32 | N/N |
| 40 | 85 | 57.36 | 9,35 | 8.89 | Calcium-binding protein | 14 | 12 | 70 | N/N |
| 41 | 62 | 57.14 | 6,82 | 4.54 | Isomerase | 27 | 34 | 29 | N/N |
| 42 | 157 | 59.91 | 17,27 | 9.17 | Uncharacterized protein | 25 | 27 | 29 | N/N |
| 43 | 315 | 62.44 | 34,65 | 6.03 | Spore photoproduct lyase | 98 | 39 | 12 | N/N |
| 44 | 116 | 54.41 | 12,76 | 4.87 | Protein parc with unknown function | 29 | 16 | 33 | N/N |
| 45 | 129 | 58.97 | 14,19 | 9.44 | Succinate dehydrogenase/fumarate reductase flavoprotein, catalytic domain | 69 | 26 | 18 | N/N |
| 46 | 86 | 49.04 | 9,46 | 5.76 | Peptidase family u32 with unknown function | 28 | 36 | 26 | N/N |
| 47 | 330 | 61.02 | 36,3 | 10.33 | Tyrosine recombinase XerH | 100 | 77 | 18 | N/N |
| 48 | 88 | 51.68 | 9,68 | 4.94 | Protein p31 | 33 | 23 | 40 | Y/N |
| 49 | 228 | 59.24 | 25,08 | 6.96 | DNA binding protein | 100 | 95 | 20 | N/N |
| 50 | 163 | 52.64 | 17,93 | 6.65 | TerB-like | 37 | 25 | 44 | N/N |
| 51 | 186 | 52.94 | 20,46 | 4.54 | Chaperone | 45 | 22 | 32 | N/N |
| 52 | 129 | 56.15 | 14,19 | 5.62 | Uncharacterized protein | 24 | 22 | 18 | N/N |
| 53 | 234 | 63.40 | 25,74 | 9.61 | Hypothetical protein pa3008 | 100 | 47 | 23 | N/N |
| 54 | 71 | 50 | 7,81 | 4.73 | Hydrolase | 37 | 76 | 22 | N/N |
| 55 | 215 | 58.64 | 23,65 | 9.39 | DNA ligase | 100 | 97 | 24 | N/N |
| 56 | 126 | 65.09 | 13,86 | 5.46 | Hydrolase | 97 | 54 | 19 | N/N |
| 57 | 144 | 61.37 | 15,84 | 5.07 | Minor ampullate spidroin | 56 | 55 | 14 | N/Y |
| 58 | 94 | 63.85 | 10,34 | 8.54 | TIR domain | 11 | 53 | 22 | Y/Y |
| 59 | 147 | 58.33 | 16,17 | 5.82 | HNH endonuclease | 92 | 41 | 21 | N/N |
| 60 | 114 | 62.31 | 12,54 | 4.93 | Ligase | 33 | 25 | 21 | N/N |
| 61 | 150 | 61.81 | 16,5 | 8.60 | Endolysin,claudin-4 | 100 | 98 | 28 | N/N |
| 62 | 82 | 61.4 | 9,02 | 8.16 | Signaling protein tnfaip3-interacting protein 2 | 71 | 44 | 31 | Y/N |
| 63 | 156 | 53.9 | 17,16 | 8.80 | Alginate and motility regulator z | 100 | 31 | 44 | N/N |
| 64 | 59 | 56.1 | 6,49 | 6.67 | Alginate and motility regulator z | 100 | 80 | 55 | N/N |
| 65 | 289 | 60.22 | 31,79 | 8.77 | "Winged helix" DNA-binding domain | 33 | 8 | 21 | N/N |
| 66 | 243 | 60.92 | 26,73 | 6.53 | Iron-dependent repressor IdeR | 94 | 26 | 16 | N/N |
| 67 | 225 | 61.06 | 24,75 | 5.52 | Sulfite reductase | 61 | 22 | 28 | N/N |
| 68 | 274 | 54.54 | 30,14 | 5.57 | d-mycarose 3-c-methyltransferase | 100 | 73 | 16 | N/N |
| 69 | 62 | 44.44 | 6,82 | 3.96 | Transcriptional activator tipa-s | 19 | 40 | 20 | N/N |
| 70 | 352 | 57.03 | 38,72 | 6.57 | Ribonuclease Rh-like | 66 | 8 | 28 | N/N |
| 71 | 189 | 61.13 | 20,79 | 5.57 | Iron binding protein lipoprotein | 36 | 55 | 8 | N/N |
| 72 | 387 | 63.48 | 42,57 | 4.55 | Baseplate wedge protein gp6 | 100 | 90 | 13 | N/N |
| 73 | 148 | 61.96 | 16,28 | 3.96 | gpW/gp25-like, Contains phage tail lysozyme | 88 | 53 | 18 | N/N |
| 74 | 172 | 59.53 | 18,92 | 5.07 | Baseplate assembly protein v | 100 | 74 | 16 | N/N |
| 75 | 383 | 60.85 | 42,13 | 4.90 | Tail protein | 100 | 82 | 17 | N/N |
| 76 | 520 | 60.46 | 57,2 | 9.03 | Hydrolase morphogenesis protein 1 | 100 | 25 | 20 | N/N |
| 77 | 474 | 62.80 | 52,14 | 5.02 | Viral hypothetical protein | 97 | 22 | 17 | N/N |
| 78 | 186 | 60.42 | 20,46 | 5 | Chaperone | 48 | 11 | 19 | N/N |
| 79 | 124 | 57.33 | 13,64 | 4.75 | 2Fe-2S ferredoxin-like | 36 | 15 | 11 | N/N |
| 80 | 496 | 63.58 | 54,56 | 5.04 | Uncharacterized protein dsy3957 | 100 | 75 | 16 | N/N |
| 81 | 62 | 61.90 | 6,82 | 5.61 | Carboxypeptidase inhibitor | 26 | 39 | 39 | N/N |
| 82 | 199 | 62 | 21,89 | 4.59 | Phage tail protein-like | 95 | 60 | 16 | N/N |
| 83 | 113 | 58.47 | 12,43 | 4.49 | Phage tail proteins | 100 | 85 | 28 | N/N |
| 84 | 354 | 60.75 | 38,94 | 5.15 | Putative capsid protein of prophage | 100 | 92 | 11 | N/N |
| 85 | 309 | 66.88 | 33,99 | 4.99 | Head decoration protein D (gpD, major capsid protein D) | 95 | 20 | 11 | N/N |
| 86 | 205 | 64.40 | 22,55 | 5.50 | Lambda repressor-like DNA-binding domains | 2 | 9 | >5 | N/Y |
| 87 | 292 | 59.95 | 32,12 | 4.72 | Protease 4 | 100 | 90 | 19 | N/N |
| 88 | 562 | 60.98 | 61,82 | 5.21 | Portal protein | 99 | 65 | 9 | N/N |
| 89 | 81 | 57.72 | 8,91 | 9.77 | Head-to-tail joining protein W, gpW | 98 | 65 | 32 | N/N |
| 90 | 416 | 62.90 | 45,76 | 8.68 | Formate dehydrogenase protein FdhE-like | 94 | 12 | 24 | N/N |

^a^kDa were calculated based on aa sequence; PDB, protein data bank; pI, isoelectric point; Con, confidence; Cov, coverage, Id, identity;TMD, Transmembrane domain; SP, signal Peptide; Y, present of TMD/SP; N, not present of TMD/SP.


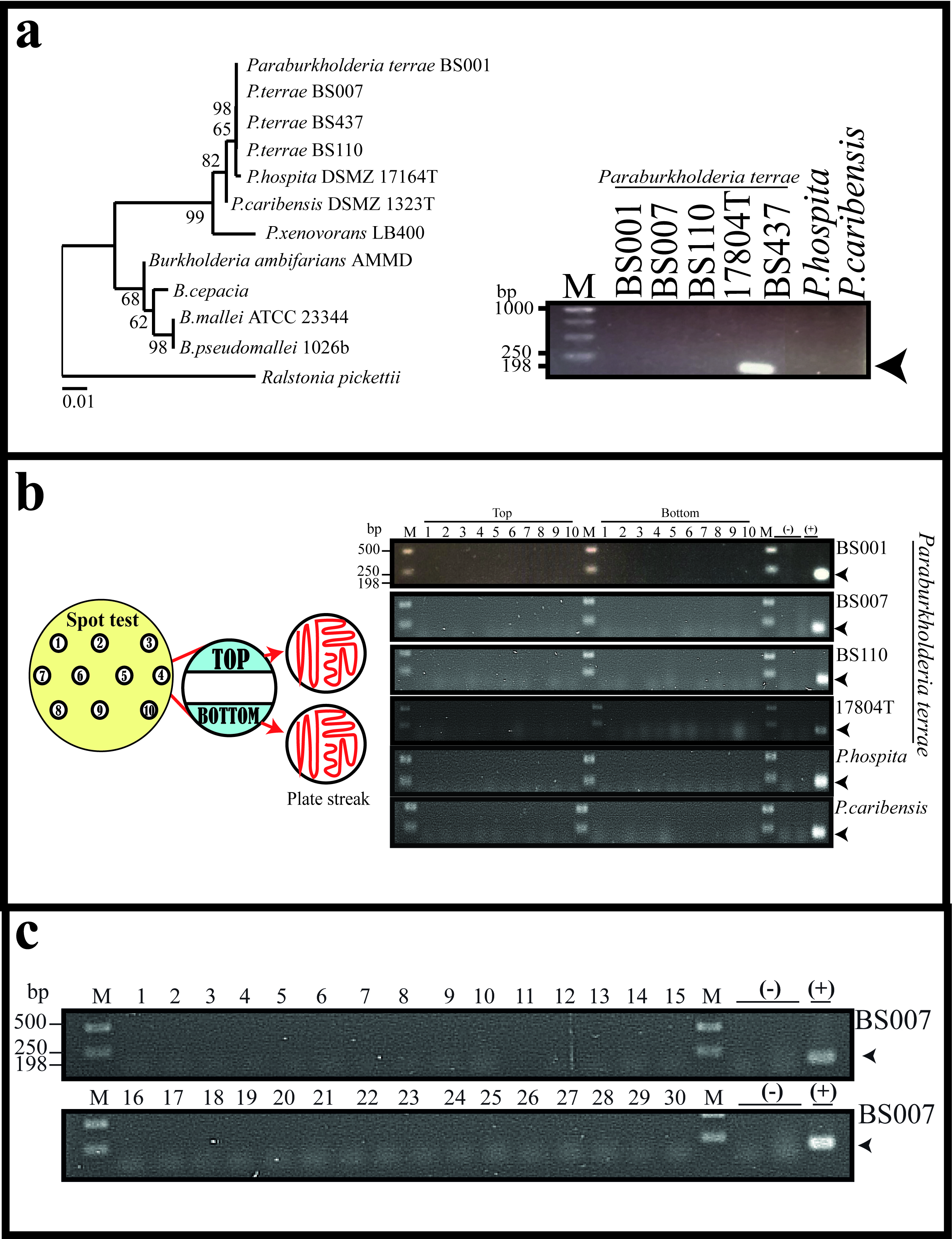


**Figure S1. The examination of the presence of prophage within indicator hosts.** To address the integration events, the PCR-based tests were conducted. **(a)** Using the specific primer sets for phage ɸ437 major capsid protein. **(b)** The induced lysate were spotted onto the plates to later streak onto new plates and tested for the present of phage ɸ437 major capsid protein. Total of 20 colonies from each spots and indicator bacteria were tested. For negative control, colonies from pure culture of corresponding indicator bacteria strains were use, along with *E.coli* E12. For positive control, phage DNA and lysate were used. **(c)** The total of 30 more colonies from of *P.terrae* were further investigated.


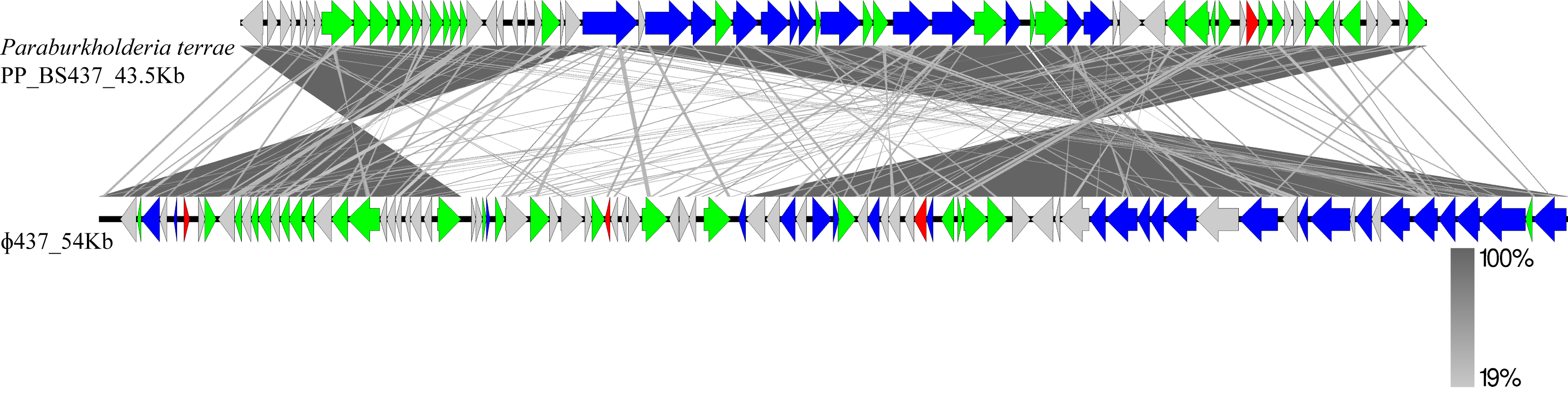


**Figure S2. Comparison of initially-identified PP region in host BS437 genome (PP_BS437) and sequenced of induced ɸ437.** Comparison percentage was generated using BLAST+ 2.4.0 (tBLASTx with cutoff value 10^-3^) and map comparison figures were created with Easyfig as indicated in material and methods. Gene similarity percentage is indicated in gray scale bar. Red arrows indicate phage lysis and lysogenic genes; blue arrows indicate phage structural genes (tail, capsid and fiber); green arrows indicate replication, recombination, repressor and phage related genes; gray arrows indicate hypothetical proteins.

**
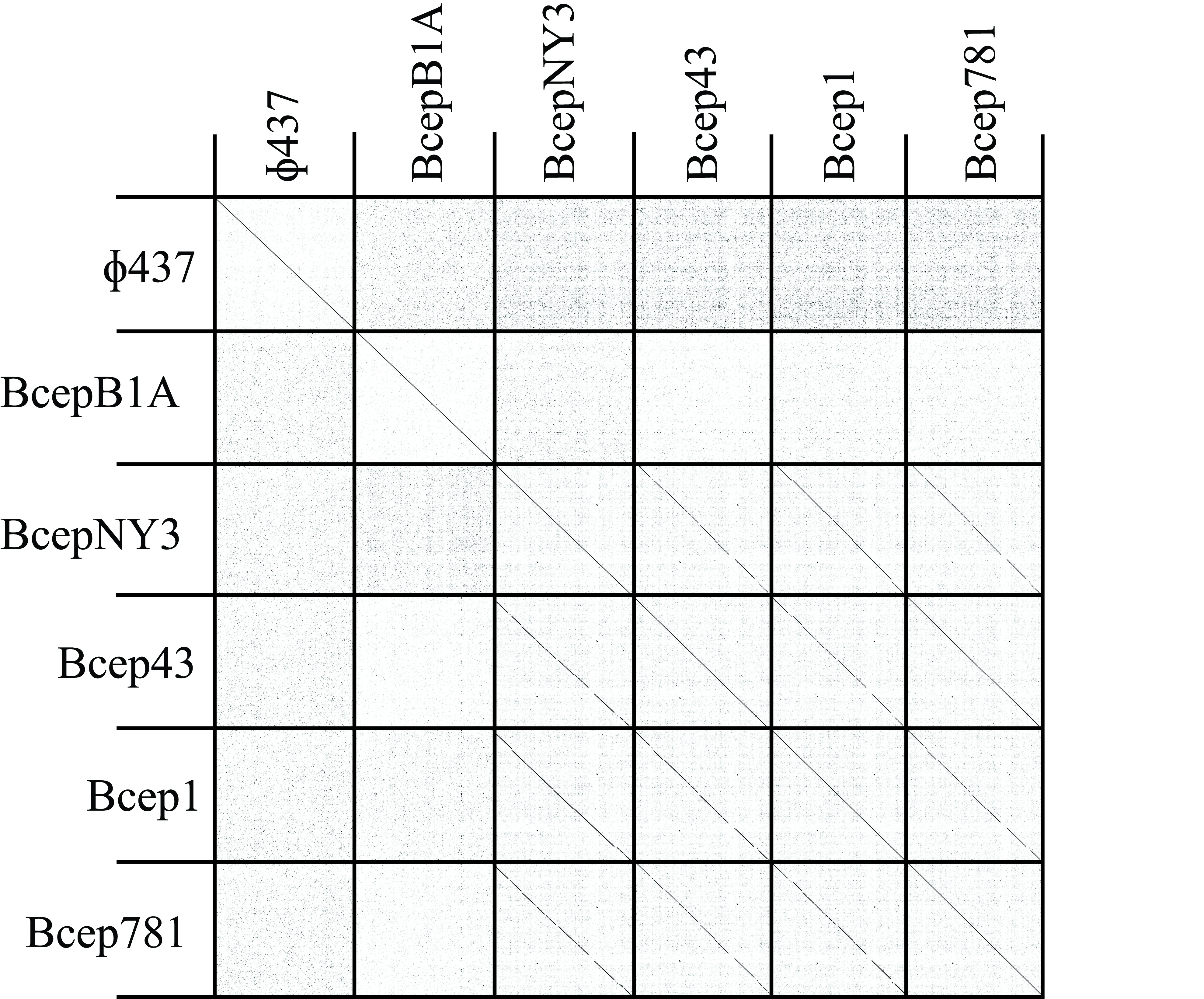
**

**Figure S3. Nucleotide dot-plot analysis.** The fasta nucleotide comparisons of ɸ437 and other *Paraburkholderia* phages were compared using Gepard program^65^ with default parameters (see Materials and Methods), indicated by the diagonal lines.
